# Food Environment, Food Access, and Disparities in Pancreatic Cancer Outcomes: A Narrative Review of Evidence Gaps and Future Directions

**DOI:** 10.3390/nu18213635

**Published:** 2026-10-31

**Authors:** Christopher Liu, Charlotte Burch, Vashti Bandy, Anna Gibson, Vignesh Vudatha, Devon Freudenberger, Jacob Lambdin, Kelly Herremans, Andrea Riner, Leopoldo Fernandez, Jose Trevino

**Affiliations:** 1 Department of Orthopaedic Surgery, University of California San Diego School of Medicine, La Jolla, CA 92037, USA; 2 Department of Surgery, Virginia Commonwealth University School of Medicine, Richmond, VA 23298, USA; 3 Department of Surgery, University of North Carolina School of Medicine, Chapel Hill, NC 27599, USA; 4 Department of Surgery, Emory University, Atlanta, GA 30322, USA

**Keywords:** food environment, food desert, food swamp, food insecurity, pancreatic cancer, pancreatic ductal adenocarcinoma, nutrition, social determinants of health, health disparities

## Abstract

Pancreatic cancer has among the poorest survival of any malignancy, with substantial disparities in outcomes. Nutritional status is associated with treatment tolerance, postoperative complications, and survival in pancreatic cancer, while dietary factors have also been associated with pancreatic cancer risk. The food environment, including access to and the relative availability of healthy and unhealthy food retailers, represents a potentially modifiable structural determinant of health. In this narrative review, we summarize evidence on nutrition and pancreatic cancer and on food environment and cancer outcomes. Few studies have examined food environment in pancreatic cancer, and findings are inconsistent. A large U.S. study using a composite food environment measure found associations with later-stage presentation and modestly worse survival, and a county-level analysis reported higher pancreatic cancer mortality in food swamps. Studies examining food desert residence, however, have not demonstrated associations with pancreatic cancer incidence or survival. Multi-cancer studies, including pancreatic cancer, report associations between poor food environments and cancer outcomes but generally do not report pancreas-specific results. Current evidence linking food environment to pancreatic cancer outcomes is limited and extrapolated from other cancers and chronic conditions, comes entirely from the United States, and relies on area-level exposure measures. We describe community interventions to improve food access, none yet evaluated for cancer outcomes, and propose a research agenda to address these gaps.

## 1. Introduction

Pancreatic cancer is one of the most lethal malignancies, accounting for an estimated 490,786 deaths worldwide in 2024 [1]. In the United States, pancreatic cancer, of which pancreatic ductal adenocarcinoma (PDAC) is the most common subtype, carries a five-year relative survival of 13% for patients diagnosed during 2015–2021, and its incidence rose by approximately 1% annually from 1998 to 2022 [2]. Despite advances in systemic therapy, surgical resection remains the only potentially curative treatment; however, the majority of patients are ineligible for surgery due to locally advanced or metastatic disease at the time of diagnosis [2]. Even among those who are eligible to undergo resection, long-term survival prospects remain dismal.

Understanding patient characteristics associated with inequalities in pancreatic cancer outcomes is critical to improving cancer outcomes and promoting health equity. Factors such as race and ethnicity, insurance status, age, genetic predisposition, geographic location, and socioeconomic status have all been identified as contributors to differences in pancreatic cancer presentation and outcomes [3,4]. Preventable differences in health outcomes, commonly referred to as healthcare disparities, are pervasive and can impact disease prevalence, access to treatment, and overall mortality. While elucidating the mechanisms underlying these known health disparities is paramount, continuing efforts to explore previously underrecognized disparities is imperative to achieving equitable care. The *food environment*, described by the Food and Agriculture Organization of the United Nations as “the collective physical, economic, policy and sociocultural surroundings, opportunities and conditions that influence people’s food and beverage choices and nutritional status”, represents one such underrecognized determinant [5].

Diet and nutrition are key contributors to a patient’s health and are shaped by a complex interplay of behavioral, socioeconomic, psychological, geographical, and cultural elements [6,7,8,9]. Emerging evidence has highlighted the significant role of diet and nutrition across the continuum of pancreatic cancer from primary prevention to treatment response [10,11,12]. Socioeconomic and geographic barriers may limit access to healthy foods that promote optimal nutrition, potentially contributing to inequities in pancreatic cancer outcomes [13].

A recent scoping review of the retail food environment and cancer outcomes identified 21 studies, all from the United States, of which only four included pancreatic cancer and only one, an analysis of incidence, examined it alone [14]. To our knowledge, our review is the first to focus on the food environment in pancreatic cancer, integrating evidence on the disease’s distinctive nutritional vulnerability with the emerging food environment literature. This narrative review addresses three questions: (1) how nutritional status and diet relate to pancreatic cancer outcomes; (2) what is known about the association of the food environment, particularly food deserts and food swamps, with dietary quality and health outcomes and how this could influence pancreatic cancer outcomes; and (3) which interventions to improve the food environment exist, and what evidence would be needed to evaluate their effect on pancreatic cancer outcomes. Direct evidence linking the food environment to pancreatic cancer is scarce. We therefore state explicitly where findings derive from pancreatic cancer populations and where they are extrapolated from other cancers and chronic conditions. Because nearly all available research on the food environment and cancer outcomes comes from the United States, the review focuses on the US context; the transferability of these constructs to other health systems is considered in Section 11.

**Figure 1:**
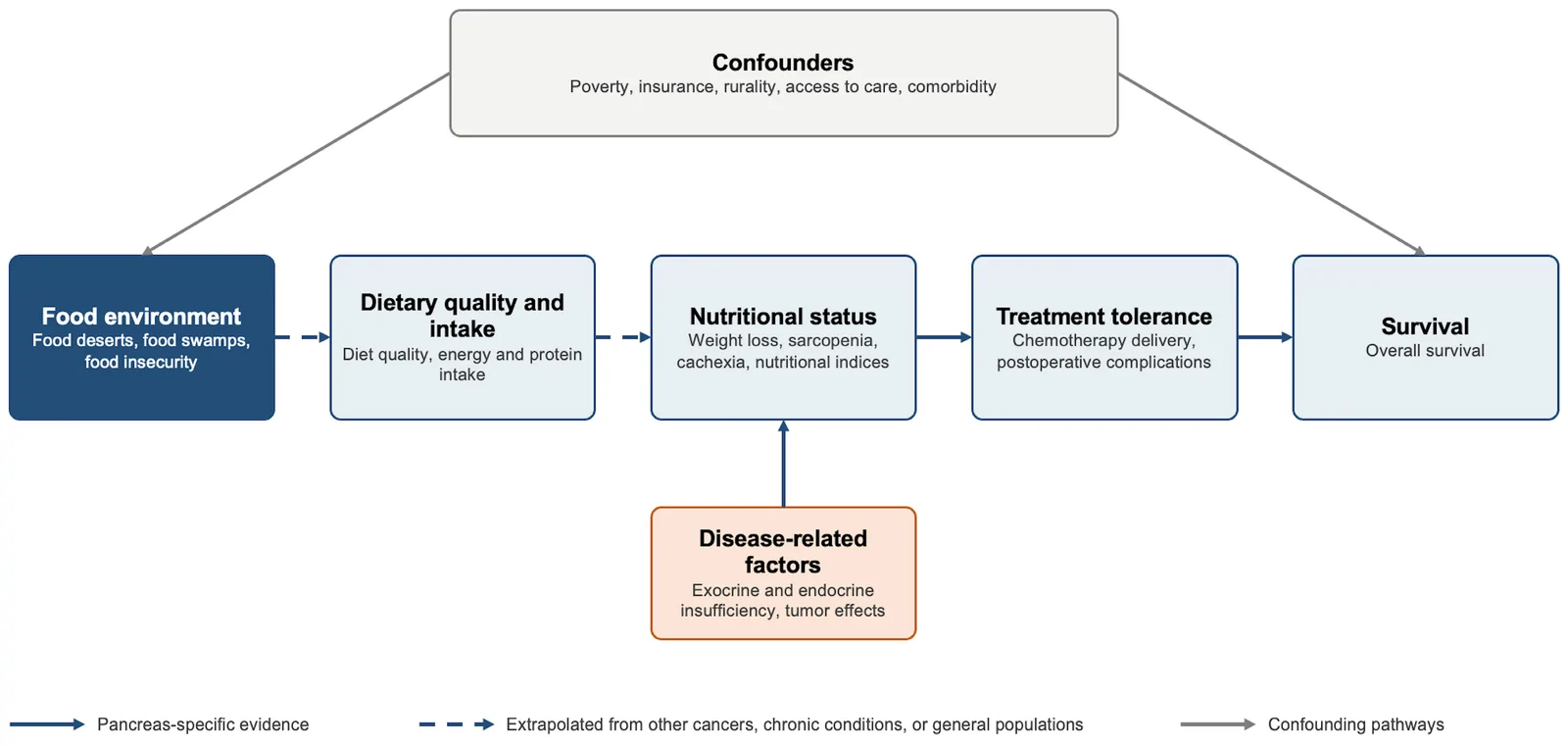
Proposed pathways linking the food environment to pancreatic cancer outcomes.

In addition to the food environment, defined above, several related but distinct concepts are used throughout this review. *Food access* is an individual’s or community’s ability to reach and afford healthy food, shaped by proximity, transportation, and income [15]. As defined by the USDA, *food insecurity* is “a household-level economic and social condition of limited or uncertain access to adequate food” [16]. *Dietary quality* describes how closely actual intake aligns with dietary recommendations, as measured by indices such as the Healthy Eating Index [17]. *Nutritional status* is the physiological state resulting from nutrient intake and utilization [18], assessed through weight change, body composition, and clinical nutritional indices [18,19]; its impairment, malnutrition, is characterized by altered body composition and diminished function and clinical outcomes [20]. Conceptual frameworks propose that the food environment shapes food choices and diets and, through them, nutritional status (Figure 1), although empirical evidence linking food environments to diet and health outcomes, including cancer, remains mixed [14,21,22,23,24].

## 2. Methods

This narrative review was informed by a literature search of PubMed and Google Scholar conducted in September 2026, with no restriction on publication date. Search terms were grouped into pancreatic cancer terms (“pancreatic neoplasms”, “pancreatic cancer”, “pancreatic ductal adenocarcinoma”); nutrition terms (“malnutrition”, “nutritional status”, “cachexia”, “nutritional support”, “prehabilitation”, “pancreatic enzyme replacement therapy”, “diet quality”, “dietary patterns”, “Healthy Eating Index”, “dietary inflammatory index”); food environment terms (“food environment”, “food desert”, “food swamp”, “food insecurity”, “food access”, “retail food environment index”); outcome terms (“neoplasms”, “cancer”, “postoperative complications”, “surgical outcomes”, “obesity”, “chronic disease”); and intervention terms (“supermarket”, “corner store”, “farmers market”, “mobile market”, “food voucher”, “medically tailored meals”). Pancreatic cancer terms were combined with nutrition terms (1600 records in PubMed) and with food environment terms (2 records in PubMed). Because few studies addressed pancreatic cancer and the food environment directly, food environment terms were also combined with outcome terms (4288 records in PubMed) and with intervention terms (548 records in PubMed). Google Scholar was searched using the same terms to supplement PubMed. Only English-language articles were included. Ongoing trials were identified through targeted Google searches, and the reference list of a recent scoping review of the food environment and cancer was hand-searched. To describe community and policy initiatives not yet evaluated in the peer-reviewed literature, targeted Google searches identified reports, municipal codes, and news coverage; these sources are cited only to describe the existence and design of such programs, not their effectiveness.

For searches returning fewer than 100 records, all titles were screened; for larger searches, the first 100–200 results ranked by relevance were screened. Studies were selected for relevance to the review questions, with the aim of providing a comprehensive overview of the evidence rather than an exhaustive account of any single topic. Studies of other cancers and chronic conditions were included where they informed plausible mechanisms and are identified as such in the text. Peer-reviewed original studies, systematic reviews, and meta-analyses were prioritized; conference abstracts were only included when they reported pancreas-specific food environment findings, given the scarcity of such data, and are identified as abstracts in the text and Table 1. Articles were screened and selected by C.L. and C.B. As a narrative review, this work did not follow a formal systematic review protocol, and no quantitative synthesis was performed.

## 3. Nutritional Status of Pancreatic Cancer Patients

The disruption of pancreatic endocrine and exocrine function in patients with pancreatic cancer is directly detrimental to the physiologic processes involved in digestion and nutritional status [35]. This impairs the production of digestive enzymes and the regulation of metabolism through insulin and glucagon, with exocrine dysfunction demonstrated by pancreatic exocrine insufficiency (PEI) that commonly develops from both tumor-related destruction or obstruction of pancreatic exocrine tissue and surgical resection [36]. These dysfunctions frequently lead to clinical manifestations including steatorrhea, weight loss, and new-onset diabetes [35,36]. For exocrine dysfunction, pancreatic enzyme replacement therapy (PERT) is the principal treatment [37]. In addition, cancer-associated cachexia, a syndrome of progressive muscle and weight loss driven by tumor-related inflammation and metabolic changes, further compromises nutritional status in this patient population, making nutritional deterioration difficult to reverse with dietary intake alone [38,39]. The manifestations of these disease processes are profound even before diagnosis, with more than 60% of patients experiencing significant weight loss (greater than 5% of baseline body weight) in the 6 months prior to diagnosis and 40% losing 10% or more [40,41].

The treatment of pancreatic cancer can further exacerbate these physiologic disturbances. Systemic therapy may contribute through anorexia, gastrointestinal toxicity, fatigue, and treatment-related reductions in dietary intake, as shown by studies illustrating how neoadjuvant chemotherapy reduces nutritional status scores and is associated with worse prognostic nutritional indices [42,43,44]. Surgical interventions, including pancreaticoduodenectomy and distal pancreatectomy, also place patients at higher risk of moderate to severe malnutrition [45]. Resection increases risk of PEI, delayed gastric emptying (DGE), and reduced oral intake in the perioperative period [46]. These altered nutritional states may require management with PERT, motility agents, or supplemental nutrition [46]. The type of surgical resection may influence nutritional outcomes, as patients undergoing pylorus-preserving procedures have demonstrated significantly greater postoperative albumin levels and less visceral fat loss compared to patients undergoing pylorus-resecting pancreaticoduodenectomies; however, this finding is not consistent across studies [46,47].

These disease and treatment-related losses occur against a background that differs across patients. Individuals with limited access to affordable nutrient-dense food, or those experiencing food insecurity, may begin treatment with poorer dietary quality and fewer reserves, and may have less ability to follow dietary recommendations such as small, frequent high-protein meals or PERT [48]. Socioeconomic barriers may further limit access to nutritional services [49]. Whether these factors measurably worsen nutritional status in pancreatic cancer has not been studied directly, a gap considered in Section 10.

## 4. Effects of Nutrition on Survival and Surgical Outcomes in Pancreatic Cancer

Nutritional and inflammatory status, assessed using measures such as body weight and composite indices incorporating serum albumin, C-reactive protein (CRP), lymphocyte count, and total cholesterol, is consistently associated with outcomes in pancreatic cancer [50,51,52,53,54,55]. Because albumin and CRP are strongly influenced by systemic inflammation, they are better regarded as markers of combined nutritional and inflammatory status rather than as direct measures of nutrition. Poor scores on nutritional indices, such as the Geriatric Nutritional Risk Index (GNRI), Controlling Nutritional Status (CONUT), and Prognostic Nutritional Index (PNI), and malnutrition identified by the Subjective Global Assessment have been linked to worse survival and postoperative complications in pancreatic cancer patients [10,50,54,55,56,57,58].

Malnutrition-associated phenotypes, including cancer cachexia and sarcopenia, have also been associated with poorer survival in pancreatic cancer, although the strength of evidence differs by measure. Sarcopenia has been consistently associated with worse survival: a 2019 meta-analysis found a 95% higher risk of death in patients with sarcopenia compared with those without, a pattern confirmed in a 2025 meta-analysis that also demonstrated worse progression-free survival [59,60]. For weight loss, the threshold appears to matter: weight loss exceeding 10% has been associated with shorter survival, whereas loss meeting the broader 5% cachexia threshold has not [40,41].

Poor nutritional status may also compromise the delivery of cancer treatment. Sarcopenia and early muscle loss during chemotherapy have been associated with dose-limiting toxicity in prior research, although a systematic review found this association in only a third of studies [61,62]. Among patients undergoing surgical resection, poor preoperative nutritional status is associated with increased rates of complications. For example, patients undergoing pancreaticoduodenectomy or pancreatectomy with worse CONUT scores have been found to experience higher rates of both overall and severe postoperative complications compared to those with more optimal scores [63]. Additionally, lower preoperative PNI scores have been linked to higher rates of postoperative complications, particularly pancreatic fistulas, in patients undergoing pancreaticoduodenectomy [10]. Furthermore, in a national US inpatient database, patients coded with a protein-energy malnutrition diagnosis had higher in-hospital mortality and more complications across multiple organ systems following pancreaticoduodenectomy; however, these data predate the 2015 transition to ICD-10 coding, and administrative codes tend to underreport malnutrition [64].

## 5. Nutritional Intervention in Pancreatic Cancer

Research investigating therapies aimed at improving the nutritional status of patients with pancreatic cancer supports the integration of nutritional care into standard treatment protocols. This approach is consistent with international guidelines recommending early nutritional assessment, individualized dietary counseling, and nutritional support throughout cancer treatment for patients at risk of malnutrition [42]. These interventions vary in both modality and timing relative to the course of cancer therapy, ranging from prehabilitation before surgery to ongoing nutritional management during chemotherapy and postoperative recovery. Studies of nutritional interventions have primarily demonstrated effects on intermediate outcomes, including body weight, body composition, functional status, and postoperative complications, while evidence for improved survival remains limited and largely derived from small or heterogeneous studies [65].

Preoperative interventions studied include nutrient supplementation, such as eicosapentaenoic acid (EPA), and diet-focused approaches, such as dietary modification, enteral nutrition, and parenteral nutrition. These have been associated with improvements in skeletal muscle area, body weight, and handgrip strength, although individual studies have assessed different outcomes [65,66,67]. Multimodal prehabilitation, incorporating nutritional support with exercise and, in some programs, psychosocial interventions, has shown potential to mitigate nutritional deterioration, decrease patient frailty, reduce length of stay, and improve functional capacity prior to pancreatic surgery, largely in mixed hepatopancreatobiliary cohorts [68,69,70]. In a randomized trial of patients undergoing major hepatobiliary and pancreatic cancer surgery, both conventional and goal-directed multimodal prehabilitation increased 6 min walk distance by approximately 30 m (27 and 31 m, respectively), a magnitude within the range reported as clinically important for this measure [71,72]. However, studies are heterogeneous and findings regarding postoperative complications are inconsistent [68,69]. Nutritional assessment and appropriate nutritional support are nonetheless recommended components of perioperative care in pancreatic surgery [46].

The period during which patients undergo chemotherapy represents a potentially valuable, yet underexplored, window for nutritional optimization. In a randomized trial by Kim et al., patients with pancreatic or bile duct cancers (29% pancreatic) received 8 weeks of either a standard diet or supplemented diet that provided additional calories, protein, and fat [73]. The supplemented group gained fat-free mass, skeletal muscle mass, and body cell mass, whereas the control group showed declines within the same parameters, and patient-reported outcomes significantly improved with supplementation. More recently, a single-center randomized trial of 60 patients with advanced pancreatic cancer found that early elemental diet supplementation during chemotherapy attenuated BMI loss and preserved handgrip strength, with longer overall survival than usual diet (21.4 vs. 12.8 months; HR 0.55, 95% CI 0.31–0.98) [12]. Given the small sample size, this survival signal remains preliminary, but it suggests that nutritional support during systemic therapy may have effects beyond weight preservation.

For patients with PEI, PERT is an important component of nutritional management. However, evidence for its effect on nutritional and oncologic outcomes in pancreatic cancer remains limited. In a randomized trial of patients with unresectable pancreatic cancer receiving chemotherapy, pancrelipase did not significantly improve BMI or other nutritional markers, and the observed difference in overall survival did not reach statistical significance [74]; a meta-analysis of randomized trials likewise found no difference in survival [75].

Evidence for specific postoperative nutritional interventions in pancreatic cancer remains limited. In the NUTRIWHI randomized trial, nutritionally at-risk patients undergoing pancreaticoduodenectomy received early supplemental enteral nutrition plus oral feeding or oral feeding alone. Among 118 analyzed patients, early enteral nutrition reduced the 90-day Comprehensive Complication Index (25.5 vs. 35.8; *p* = 0.02), although individual complications, including pancreatic fistula, delayed gastric emptying, and infection did not differ [76]. These findings are consistent with perioperative guidelines favoring early oral or enteral nutrition when feasible [77]. Smaller studies have also reported that high-fat, high-energy ketogenic diets increase energy intake and that high-fat oral nutritional supplements increase caloric intake and improve nutritional risk index scores [78,79]. Together, these findings support continued investigation of targeted postoperative nutritional support.

Access to structured prehabilitation and multidisciplinary nutritional services may also be inconsistent. A 2026 survey of cancer-surgery prehabilitation services across the UK National Health Service found substantial variation in service availability and characteristics, with only 65% of responding organizations reporting an eligible prehabilitation service [80]. Other research has reported that, even in a universal health system, 44% of patients with pancreatic cancer and cachexia had no dietetic consultation [40]. Financial, geographic, and transportation barriers may further limit access to dietetic services, oral nutritional supplements, and other nutritional interventions, although these relationships have not been well-studied in pancreatic cancer and represent an important gap (Section 10).

## 6. Diet and Pancreatic Cancer

International guidelines recommend that patients with cancer consume 25–30 kcal/kg/day of energy and more than 1.0 g/kg/day of protein, up to 1.5 g/kg/day where possible [42]. Many patients with pancreatic cancer fall short of these targets. In a Dutch multicenter cohort, reduced food intake was reported by 53% of patients undergoing surgery and 79% of those receiving palliative chemotherapy, and protein intake was insufficient in 66% and 76%, respectively [40].

Limited evidence suggests that dietary intake during treatment may be associated with outcomes. In a prospective cohort of patients with unresectable pancreatic cancer receiving chemotherapy, protein intake below 1.1 g/kg/day after treatment initiation was independently associated with shorter overall survival [81]. In a cohort of patients with advanced disease, a plant-rich dietary pattern was associated with better chemotherapy response and longer survival than a high-fat Western pattern [82]. However, both studies were small and observational, and lower intake may partly reflect more advanced disease rather than cause poorer outcomes. Evidence that improving diet during treatment improves survival is limited and inconsistent [83], and the quality of diet after diagnosis, beyond energy and protein intake, remains largely unexplored [84].

Most research on diet and pancreatic cancer instead concerns risk of developing the disease. Excess body fatness is an established cause of pancreatic cancer, and in observational studies, healthier dietary patterns have generally been associated with lower risk [85,86]. In the NIH-AARP cohort of more than 500,000 adults, those with the highest Healthy Eating Index scores had a 15% lower risk than those with the lowest (HR 0.85, 95% CI 0.74–0.97), and a subsequent analysis found similar reductions for the Healthy Eating Index-2015, Mediterranean, and DASH dietary patterns (HRs 0.82–0.86), though not for the Alternative Healthy Eating Index [87,88]. Similar associations have been reported in Asian and Australian cohorts and European case–control studies, although not consistently [89,90,91,92]. Higher dietary antioxidant capacity has been associated with lower pancreatic cancer incidence and mortality in population-based studies, and diets with higher inflammatory potential with increased risk, including an 11% higher risk per standard-deviation increase in dietary inflammatory score in the multinational EPIC cohort (HR 1.11, 95% CI 1.02–1.22) [93,94,95,96,97]. These findings are relevant to prevention and to disparities in pancreatic cancer incidence, but they cannot be assumed to apply to patients already diagnosed. Notably, none of the treatment-period studies described above measured patients’ food environment or food access, so whether these factors contribute to inadequate intake during treatment is unknown, a gap addressed in Section 10.

## 7. Food Deserts

Food deserts are generally defined as low-income areas in which a substantial proportion of residents have limited access to healthy and affordable food vendors, although definitions vary across studies [98] (Table 2). Food deserts may represent one important mechanism through which socioeconomic and geographic disadvantage influences dietary intake and, potentially, health outcomes in pancreatic cancer. However, few studies have examined food deserts in pancreatic cancer, as discussed at the end of this section. The evidence below therefore comes largely from general populations, other chronic conditions, and other cancers and is presented as indirect evidence of mechanisms that may plausibly apply to pancreatic cancer.

In a cross-sectional analysis of a nationwide U.S. cohort, food desert residence was independently associated with poor diet quality, defined as scoring in the lowest quartile of the 2020 American Cancer Society (ACS) guidelines for cancer prevention. Individuals living in food deserts had a 17% higher risk of poor dietary quality and lower scores for vegetable, fruit, sugar-sweetened beverages, and red/processed meat [48]. Similarly, in the REGARDS cohort, residents of food desert census tracts were more likely to adhere to a Southern dietary pattern characterized by fried foods, added fats, eggs and egg dishes, sugar-sweetened beverages, organ meats, and processed meats [99]. In both studies, diet quality was also associated with education, income, and race and ethnicity, which are concentrated in food desert areas, making it difficult to isolate the independent contribution of food access.

The availability of healthy food may also influence the effectiveness of dietary interventions. Wedick et al. found that proximity to healthy food retailers modified the impact of a dietary behavioral intervention in overweight and obese adults: for each additional mile a participant lived from a healthy food store, improvements in fiber intake were 1.07 g/day less and improvements in fruit and vegetable intake were 0.19 servings/day less [100]. These findings suggest that the local food environment may influence the effectiveness of nutritional interventions, a consideration potentially relevant to nutritional support in pancreatic cancer, although this relationship has not been directly studied in this population.

Evidence from other chronic conditions similarly suggests associations between food desert residence and adverse health outcomes. In a cross-sectional analysis of patients with chronic obstructive pulmonary disease (COPD), food desert residence was associated with lower forced expiratory volume in one second (FEV1), increased air trapping, and poorer respiratory quality of life after adjustment for demographic characteristics and income [101]. Pregnant patients with COVID-19 who required hospitalization were more likely to reside in a food desert, and among patients undergoing cardiac catheterization, food desert residence was associated with higher prevalence of myocardial infarction, prior revascularization, hypertension, and high body mass index (BMI) [102,103].

Food desert residence has also been associated with adverse surgical outcomes. Among patients undergoing total knee or hip arthroplasty, food desert residence was associated with increased length of stay and higher healthcare costs [104,105]. In patients undergoing revascularization for chronic limb-threatening ischemia, food desert residence was associated with an almost threefold increase in 30-day readmission and higher rates of wound complications [106]. Similarly, a study of patients undergoing esophagectomy found that food desert residence was associated with increased 30-day readmission after adjustment for clinical factors [107]. Because food deserts are concentrated in lower socioeconomic areas, these associations may reflect broader socioeconomic disadvantage and cannot be directly attributed to limited food access alone.

Evidence linking food deserts to cancer outcomes is emerging but inconsistent. A recent scoping review identified seven studies examining food desert residence and cancer outcomes, four of which reported significant associations with mortality or survival [14]. In a California Cancer Registry study, food desert residence was associated with poorer five-year survival among patients with breast and colorectal cancer, and a large study of endometrial, ovarian, and cervical cancers similarly found higher disease-specific mortality among patients residing in food deserts [108,109]. In contrast, other studies of gynecologic cancers and resected non-small cell lung cancer found no significant association after adjustment [110,111].

Direct evidence in pancreatic cancer is limited and inconsistent (Table 1). Only two studies have examined food desert residence in pancreatic cancer specifically. In a county-level analysis, food desert measures incorporating vehicle access were associated with higher pancreatic cancer incidence in bivariate analysis, but standard measures were not. After backward selection, none remained significant, and smoking prevalence was the only retained factor [26]. A conference abstract likewise found no association between food desert residence and pancreatic cancer survival [27]. Studies that include pancreatic cancer alongside other cancers, without reporting it separately, have found adverse associations. Among patients undergoing resection for hepatopancreatobiliary cancer, more than half of whom had pancreatic cancer, food desert residence was associated with lower odds of a textbook outcome (OR 0.86, 95% CI 0.76–0.97); this study is described further in Section 8 [31]. At the county level, counties with high food desert scores had higher odds of high mortality from obesity-related cancers, a group that includes pancreatic cancer (age-adjusted OR 1.59, 95% CI 1.29–1.94). This ecological analysis, however, cannot be attributed specifically to pancreatic cancer or establish an individual-level relationship [32].

Collectively, these findings suggest that food deserts may influence dietary quality and potentially cancer-related outcomes, but the evidence remains heterogeneous and is vulnerable to socioeconomic confounding. In pancreatic cancer, existing studies have not established whether food desert residence contributes to poorer outcomes through inadequate dietary intake, reduced access to nutritional interventions, or other mechanisms. Further research directly linking food environment, dietary intake, nutritional status, and pancreatic cancer outcomes is therefore needed (Section 10).

**Table 2:** Food environment metrics used in the studies reviewed.

Metric	Definition	Direction	Geographic Unit	Data Source	Studies in This Review
Food desert (USDA low-income/low-access)	Low-income tract where ≥500 people or ≥33% of residents live >1 mile (urban) or >10 miles (rural) from a supermarket	Binary	Census tract; zip code in [106,107,111]	USDA FARA	[26] ˜,[27,48] *,[99] ˆ,[103,106,107],[108] *,[110,111]
Food desert (USDA vehicle-access)	Tract where ≥100 households lack a vehicle and live >0.5 mile from a supermarket, or ≥500 people or ≥33% live ≥20 miles away	Binary	Census tract, aggregated to county	USDA ERS	[26]
Food desert score	Percentage of county population with low income and low access to a grocery store (>1 mile urban, >10 miles rural)	Higher = worse	County	USDA FEA	[31,32,112,113]
RFEI	Ratio of fast-food restaurants and convenience stores to supermarkets/grocery stores	Higher = worse	Residential buffer [114]; county [115]	Business listings [114]; USDA FEA [115]	[114,115]
Food swamp score (USDA)	Ratio of fast-food restaurants and convenience stores to grocery stores and farmers markets	Higher = worse	County	USDA FEA	[28],[31,32],[112] #,[113,116]
Food swamp score (BCBSA)	Ratio of fast-food outlets to grocers, adjusted for population density and income, scaled 0–10	Higher = worse	Zip code, averaged to county	BCBSA	[117]
mRFEI	Healthy food retailers as a percentage of all food retailers	Higher = better	County	InfoGroup	[33]
FEI	0–10 score combining limited access to healthy foods and food insecurity	Higher = better	County	CHR&R	[29,30]
Composite food environment score	Composite of 253 USDA FEA county variables, ranked 0–1	Higher = worse	County	USDA FEA	[25]
Activity-based retail food index	Percentage of visits to food retailers that were healthy retailers, from mobile device data	Higher = better	County	SafeGraph	[33]
Food insecurity rate †	Estimated percentage of county population that is food-insecure	Higher = worse	County	Feeding America	[34]

* Low-access criterion only. ˆ USDA Food Desert Locator (FARA predecessor). ˜ % of county tracts; ½- and 1-mile thresholds. # Supercenters counted as unhealthy and specialized stores as healthy. † Related exposure, not a food environment metric. Abbreviations: BCBSA, Blue Cross Blue Shield Association; CHR&R, County Health Rankings & Roadmaps; ERS, Economic Research Service; FARA, Food Access Research Atlas; FEA, Food Environment Atlas; FEI, Food Environment Index; mRFEI, modified Retail Food Environment Index; RFEI, Retail Food Environment Index; USDA, US Department of Agriculture.

## 8. Food Swamps

A related measure of the food environment is the food swamp, an area in which unhealthy food options outnumber healthier alternatives. The term was first introduced by Rose et al. to describe neighborhoods with an abundance of fast food and energy-dense, nutrient-poor options relative to healthier food sources [118]. Food swamps are increasingly common: between 2003 and 2023, the proportion of US census tracts classified as food swamps increased from 80.2% to 88.5%, whereas the proportion classified as food deserts changed little [119]. Like food deserts, food swamps may contribute to inequities in health outcomes by promoting dietary patterns associated with metabolic dysfunction.

Food swamps are most often quantified using the Retail Food Environment Index (RFEI) or its modified version (mRFEI), which differ in construction and direction (Table 2). The RFEI is the ratio of unhealthy food retailers, such as fast-food restaurants and convenience stores, to healthier retailers, such as supermarkets and specialty food stores, within a given area, so higher scores indicate a less healthy environment [120]. The mRFEI instead expresses healthy food retailers as a percentage of all food retailers, so higher scores indicate a healthier environment [121]. Residence in areas with higher RFEI or lower mRFEI scores has been associated with poorer dietary patterns and greater rates of adult obesity, and one national study found that food swamp measures predicted county obesity rates more strongly than food desert measures [114,115,122].

Evidence from other conditions, although not directly transferable to pancreatic cancer, suggests that food swamp residence is associated with adverse health outcomes. Phillips et al. found that adults with diabetes living in severe food swamps experienced higher rates of both all-cause and ambulatory care-sensitive hospitalizations, with approximately 50 and 19 additional hospitalizations per 1000 adults with diabetes, respectively, and larger effects in rural counties [117]. At the county level, food swamp prevalence has also been associated with higher mortality from metabolic dysfunction-associated steatotic liver disease (MASLD) [112].

Few studies have examined food swamps and cancer outcomes directly. A recent scoping review identified three studies examining food swamp exposure and cancer outcomes, all of which reported significant associations with mortality [14]. In colorectal cancer, food swamp residence has been associated with higher mortality from early-onset disease and with lower rates of textbook outcomes after surgery [113,116]. These findings provide indirect evidence that the broader food environment may influence cancer outcomes, but they cannot be assumed to apply to pancreatic cancer.

Only one study, available as a conference abstract, has examined food swamp exposure and pancreatic cancer specifically. In an ecological analysis of 94 Georgia counties, those with high food swamp scores had higher odds of high pancreatic cancer mortality from 2010 to 2020 after adjustment for age, race, poverty, and physician density (aOR 2.63, 95% CI 1.02–6.77) [28]. However, the confidence interval was wide, and population mortality reflects both incidence and survival. The model also did not adjust for smoking, which was more prevalent in high-mortality counties and was the strongest county-level predictor of pancreatic cancer incidence in a separate analysis [26]. This finding contrasts with the null results for food desert residence (Section 7), although the studies differ in design and adjustment. Other evidence comes from studies that include pancreatic cancer alongside other cancers (Table 1). In a Medicare cohort of 53,426 patients undergoing resection for hepatopancreatobiliary cancer, 57% of whom had pancreatic cancer, residence in a high food swamp county was associated with higher 90-day mortality (OR 1.74, 95% CI 1.47–2.06) and lower odds of a textbook outcome (OR 0.83, 95% CI 0.75–0.92), defined as no prolonged length of stay, complications, readmission, or death within 90 days, after adjustment for age, sex, race, county-level social vulnerability, procedure type, and comorbidity [31]. The similar association for food desert residence in the same cohort (Section 7) suggests that the two measures may capture overlapping disadvantage. However, results were not reported by cancer type, stage was unavailable, and patients with unresectable disease were excluded. County-level findings for obesity-related cancer mortality are mixed. High food swamp scores were associated with greater odds of high obesity-related cancer mortality (age-adjusted OR 1.77, 95% CI 1.43–2.19), although this association attenuated after further adjustment [32]. A national analysis, however, found no significant association for an mRFEI-type index based on retailer locations, but did find an inverse association for an index based on residents’ GPS-measured retailer visits (OR 0.82 per standard-deviation increase, 95% CI 0.75–0.90) [33]. Neither obesity-related cancer analysis can be attributed specifically to pancreatic cancer, and no study has examined whether food swamp exposure affects dietary intake, nutritional status, or treatment tolerance in patients with pancreatic cancer.

## 9. Other Food Environment and Food Access Measures in Pancreatic Cancer

Beyond food deserts and food swamps, some studies have examined broader food environment measures or food insecurity in populations that include patients with pancreatic cancer (Table 1).

In the only peer-reviewed study reporting pancreas-specific outcomes after diagnosis, 49,785 patients with pancreatic cancer were drawn from a larger gastrointestinal cancer cohort. The exposure was a county-level composite derived from the USDA Food Environment Atlas, which reflects food availability, nutrition assistance, and related social conditions. A worse score was associated with later-stage presentation of tumors in the pancreatic body and tail (OR 1.04, 95% CI 1.01–1.06), but not of tumors in the pancreatic head or at other pancreatic sites. A worse score was also associated with modestly worse overall survival across pancreatic cancer (HR 1.04, 95% CI 1.02–1.05). These associations persisted after adjustment for other social determinants of health [25].

In a North Carolina registry study of 1,292,991 patients with cancer, 30,361 of whom had pancreatic cancer, a higher county-level Food Environment Index was associated with modestly better overall survival (HR 0.983 per unit increase, 95% CI 0.979–0.988) after adjustment for stage, treatment, and county-level socioeconomic and behavioral factors [30]. This index combines limited grocery store access among low-income residents with food insecurity. The study did not report pancreas-specific estimates, although the authors found no significant interaction by anatomic site; a conference abstract from the same group reported a similar association in pancreatic cancer specifically [29].

Food insecurity, measured at the county level, was examined in a SEER-Medicare cohort of 49,882 patients with hepatopancreatobiliary cancer, 58% of whom had pancreatic cancer. Among the 6702 (13.4%) who underwent resection, residence in high-food-insecurity counties was associated with lower odds of a textbook outcome (OR 0.69, 95% CI 0.54–0.88). Residence in high-food-insecurity counties was also associated with higher mortality at 1 (HR 1.14, 95% CI 1.08–1.21), 3 (HR 1.14, 95% CI 1.08–1.20), and 5 (HR 1.07, 95% CI 1.02–1.13) years, although the 3- and 5-year associations were not significant after adjustment for county income. Pancreas-specific estimates were not reported [34].

Because these studies use different exposures and populations, and most do not report pancreas-specific estimates, their findings are not directly comparable. Whether the associations reflect nutritional pathways or broader socioeconomic and healthcare disparities remains unclear.

## 10. Interventions to Improve the Food Environment

Interventions to improve food access range from public policy reforms to community- and clinic-based programs. Some have been evaluated for effects on dietary intake, food security, or general health, with mixed results. However, to our knowledge, none of the community-level interventions has been evaluated for effects on cancer outcomes, and none has focused on patients with pancreatic cancer. They are summarized here to identify candidates for future evaluation rather than as established strategies for improving cancer outcomes.

### 10.1. Supermarket Incentives and Public Transit

Several jurisdictions use zoning or tax incentives to attract grocery stores to underserved areas, including Philadelphia’s Fresh Food Market Bonus, New York City’s Food Retail Expansion to Support Health (FRESH) program, and Prince George’s County, Maryland’s Grocery Store Tax Credit, and the federal Healthy Food Financing Initiative has funded supermarket development in food deserts nationally [123,124,125,126]. Evaluations of the impact of new supermarkets in food deserts have been mixed. In Pittsburgh, residents of a food desert that received a new supermarket reduced energy and added sugar intake relative to a comparison neighborhood, but fruit and vegetable intake and BMI did not differ, and dietary changes were unrelated to use of the store [127]; a follow-up analysis of the same cohort found fewer newly reported diagnoses of hypercholesterolemia and arthritis [128]. In contrast, supermarket openings in Philadelphia and the South Bronx under the FRESH program did not change reported fruit and vegetable consumption or children’s diets, respectively [129,130]. A systematic review of new-grocery-store interventions similarly found inconsistent evidence of effects on diet, and a national analysis estimated that equalizing grocery product availability and prices would reduce nutritional inequality by only 10%, suggesting that improving food access may have limited effects on diet quality [131,132]. Public transportation offers an alternative avenue for improving access to grocery stores, and some cities have introduced bus routes or on-demand services connecting underserved communities to grocery stores [133]. However, only about one-third of US municipalities with public transit consider food access in route planning, and, to our knowledge, the health effects of such services have not been evaluated [134].

### 10.2. Local Retailers

Interventions targeting smaller food retailers aim to increase the availability and promotion of healthy foods in corner stores. A systematic review of 16 small-store interventions found improvements in healthy food availability, purchasing, and consumption, particularly for programs combining environmental and behavioral strategies [135]. For example, New York City’s Healthy Bodegas Initiative increased sales of promoted healthy products in a substantial proportion of participating stores, and the Baltimore Healthy Stores program increased weekly sales of promoted foods in corner stores and supermarkets [136,137]. A pilot trial in four Latino food stores in North Carolina found a marginal increase in customers’ self-reported fruit and vegetable intake [138]. However, in Philadelphia’s Healthy Corner Store Initiative, a cluster randomized trial found no change in the energy content of students’ corner store purchases or in obesity measures, and a larger evaluation across 192 stores found no change in the nutritional content of purchases after one year [139,140].

### 10.3. Produce Access Programs

Farmers markets, farm stands, mobile markets, and community gardens aim to bring fresh produce directly into underserved areas [141]. A farmers market introduced in a Canadian food desert was associated with greater healthy food variety and a 12.2% lower price for a nutritious food basket, and farm stands introduced in low-income communities increased residents’ fruit and vegetable intake [142,143]. A systematic review of mobile produce markets found that their use was associated with higher fruit and vegetable consumption, although no included intervention study had a control group, and a mobile market in Pittsburgh food deserts increased vegetable intake in two of six neighborhoods [144,145]. Community gardens have been associated with psychosocial and nutritional benefits, and in the first randomized trial of community gardening, participants increased fiber intake and physical activity relative to waitlist controls [146,147,148]; however, their effect on food security remains unclear. A hydroponic garden at the Georgia Cancer Center distributes produce to patients and community residents, citing food environment-related cancer disparities as its rationale, but outcome data have not yet been reported [149,150].

### 10.4. Clinic-Based Food Interventions

Interventions delivered through health care settings are most directly relevant to patients with cancer. Outside cancer, receipt of a medically tailored meal program among patients with serious illness was associated with fewer hospital and skilled nursing admissions and lower medical spending [151]. In a randomized trial of food-insecure patients in safety-net cancer clinics, clinic-based food assistance, including a food pantry, food vouchers, and home grocery delivery, supported high treatment completion and improved food security and quality of life [152]. Screening and referral for food insecurity at preoperative surgical visits have been shown to be feasible, and trials of medically tailored meals and food assistance during active cancer treatment are ongoing [153,154,155]. None of these studies has focused on pancreatic cancer.

### 10.5. Priorities for Future Research

Three priorities would address the gaps identified in this review. First, data linkage: cancer registries and claims-based cohorts could be linked at the census-tract or address level to USDA food access data and retail food environment measures, allowing for pancreas-specific estimates adjusted for stage, treatment, insurance, and area deprivation. Because area-level measures may not reflect where patients actually shop, individual-level measures, such as activity-based indices of retailer visits, should be incorporated where possible [33]. Second, study design: prospective cohorts that screen patients for food insecurity at diagnosis, as recommended by the American Society of Clinical Oncology, for example with the two-item Hunger Vital Sign, and that measure dietary intake, nutritional status, and receipt of nutritional care, including dietetic consultation, oral nutritional supplements, and PERT, over time would allow for formal mediation analysis [156,157]. Such analysis could test whether any effect of the food environment operates through nutrition rather than access to care. Natural experiments around supermarket openings or closures, and pragmatic trials of food assistance or medically tailored meals during neoadjuvant or palliative therapy, could provide causal evidence. Third, endpoints: studies should report changes in nutritional status, treatment completion and dose intensity, receipt of surgery, postoperative textbook outcome, quality of life, and overall survival.

## 11. Limitations

Several limitations of this review must be acknowledged, relating both to the underlying evidence and to the review itself.

First, few studies have examined the food environment in pancreatic cancer populations, and most conclusions are therefore drawn by extrapolation from other cancers, chronic conditions, and general populations. The pancreas-specific studies that do exist report inconsistent findings, and three are available only as conference abstracts.

Second, people living in poor food environments are more likely to be of lower socioeconomic status, which is itself associated with limited access to transportation, housing, insurance, and healthcare, each of which independently predicts later diagnosis, lower rates of surgery, and worse survival. Whether worse outcomes observed in poor food environments are attributable to food access itself or to these related factors cannot be determined from current evidence. In the largest county-level study, for example, the association between food swamps and obesity-related cancer mortality weakened substantially after adjustment for poverty and was no longer significant after adjustment for obesity, and the composite measure used in the largest pancreas-specific study also incorporates broader social determinants [25,32].

Third, nearly all studies assigned food environment exposure at the county or census-tract level rather than for individual patients. Associations observed between areas may not hold for individuals within them (the ecological fallacy), and area-level measures do not capture where people actually shop or how they use their food environment [14]. Definitions of food deserts and food swamps also vary in thresholds, retailer classification, and geographic unit (Table 2), limiting comparison across studies.

Fourth, most studies are cross-sectional or retrospective, which limits causal inference. In pancreatic cancer, reverse causation is a particular concern: appetite loss, malabsorption, and treatment effects alter diet after diagnosis, so dietary intake measured during treatment may reflect disease severity rather than influence outcomes.

Fifth, all studies of the food environment and cancer outcomes identified were conducted in the United States, consistent with a recent scoping review [14]. Because food retail, urban design, transportation, and health insurance systems differ across countries, these findings may not generalize to other settings, particularly those with universal health coverage, although access to nutritional care may be limited even in such systems [40].

Finally, as a narrative review, this work did not follow a systematic review protocol or include formal quality assessment. Only the most relevant results of larger searches were screened, and studies were selected to illustrate the breadth of the evidence rather than to capture every study, so relevant studies may have been missed.

## 12. Conclusions

Patients with pancreatic cancer face especially high risk of nutritional impairment, driven by the disease itself and by its treatment. Diminished nutritional status is associated with decreased quality of life, increased postoperative complications, and worse overall survival. Social determinants limiting healthy food access, including residence in food deserts and food swamps, may contribute to poor nutritional status and have been linked to worse outcomes in chronic disease and other cancers. In pancreatic cancer, however, direct evidence is limited and inconsistent, and the relationship proposed in this review rests largely on extrapolation from other populations. Whether any associations are mediated primarily through nutritional status, comorbidity burden, delayed diagnosis, or healthcare access remains an open question and an important target for future investigation. Understanding how limited healthy food accessibility impacts this population is essential for informing both individual patient management and broader public health strategies. Identifying patients in areas with poor food access may allow clinicians to target those most likely to benefit from nutritional interventions and preoperative optimization, while broader initiatives expanding the availability of fresh produce may help address the underlying drivers of nutrition-related health disparities. Addressing food environment inequities is therefore a plausible but untested strategy for reducing outcome disparities in this high-risk population. Pancreas-specific studies using individual-level exposure measures, together with rigorous evaluation of food access interventions in cancer populations, are needed before such strategies can be expected to improve survival.

## Figures and Tables

**Table 1:** Studies of the food environment that include patients with pancreatic cancer.

Study	Design; Setting; Years	Pancreatic Cancer Patients	Exposure	Outcome	Pancreas-Specific Result Reported?	Main Finding
Fei-Zhang 2024 [25]	Retrospective cohort; SEER; 2013–2017	49,785 (of 287,148 GI cancers)	County composite food environment score (USDA FEA)	Late-stage presentation; Mortality; OS	Yes	Worse score: higher odds of stage IV presentation, body/tail tumors only, OR 1.04 (1.01–1.06); worse OS, HR 1.04 (1.02–1.05); 3-year mortality not significant; adjusted for age and SVI
Luberice 2025 [26]	Ecological; 157 counties in 12 SEER registries; 2012–2016	NR	% of county tracts meeting USDA food desert definitions	Incidence (above vs. below national average)	Yes	Vehicle-access measures associated in bivariate analysis; standard measures not; not significant after backward selection (smoking only significant factor)
Vudatha 2023 * [27]	Registry cohort; Virginia Cancer Registry; 2009–2018	NR	Food desert (USDA FARA; address-matched)	Survival	Yes	No association with survival
Bevel 2026 * [28]	Ecological; 94 Georgia counties; 2010–2020	NR	County food swamp score (USDA)	Mortality	Yes	High vs. low: aOR 2.63 (1.02–6.77); adjusted for age, race, poverty, physician density; not smoking
Wu 2025 *§ [29]	Retrospective cohort; North Carolina Central Cancer Registry; 2000–2022	30,361	County FEI (CHR&R)	Survival	Yes	Higher FEI associated with better survival
Wu 2025 [30]	Retrospective cohort; North Carolina Central Cancer Registry; 2000–2022	30,361 (of 1,292,991 cancers)	County FEI (CHR&R)	OS	No	All cancers: HR 0.983 per unit (0.979–0.988); adjusted for stage, treatment, and county SES and health behaviors; no significant interaction by cancer site
Khalil 2023 [31]	Retrospective cohort; Medicare; 2014–2020	30,612 (of 53,426 resected HPB cancers)	County food swamp and food desert scores (USDA FEA)	TO; Mortality; OS	No	High vs. low food swamp: TO OR 0.83 (0.75–0.92); 90-day mortality OR 1.74 (1.47–2.06). High vs. low food desert: TO OR 0.86 (0.76–0.97); 90-day mortality OR 1.52 (1.29–1.81); worse unadjusted 5-year OS for both
Bevel 2023 [32]	Ecological; 3038 US counties; 2010–2020	Included among 13 obesity-related cancers	County food swamp and food desert scores (USDA FEA)	Obesity-related cancer mortality	No	High vs. low, age-adjusted: food swamp OR 1.77 (1.43–2.19); food desert OR 1.59 (1.29–1.94). Food swamp OR 1.29 (1.03–1.63) after race and poverty adjustment, not significant after obesity adjustment
Lin 2025 [33]	Ecological; 2925 US counties; 2015–2020	Included among 13 obesity-related cancers	Location-based (mRFEI-type) and activity-based retail food indices	Obesity-related cancer mortality	No	Per SD, high vs. low mortality: location-based OR 0.90 (0.81–1.01), not significant; activity-based OR 0.82 (0.75–0.90); adjusted for demographics, SES, and food desert presence
Chinaemelum 2023 † [34]	Retrospective cohort; SEER-Medicare; 2010–2015	28,934 (of 49,882 HPB cancers; 6702 resected)	County food insecurity (Feeding America)	TO; Mortality; OS	No	High vs. low: TO OR 0.69 (0.54–0.88) and 90-day mortality OR 2.05 (1.30–3.24) among resected; 5-year mortality HR 1.07 (1.02–1.13), not significant after county income adjustment

* Conference abstract. † Measures food insecurity rather than the food environment; included as related evidence. § Abstract results section refers to colorectal cancer; title, sample size, and conclusion indicate pancreatic cancer. Values are odds ratios or hazard ratios with 95% confidence intervals. Abbreviations: CHR&R, County Health Rankings & Roadmaps; FARA, Food Access Research Atlas; FEA, Food Environment Atlas; FEI, Food Environment Index; GI, gastrointestinal; HPB, hepatopancreatobiliary; HR, hazard ratio; mRFEI, modified Retail Food Environment Index; NR, not reported; OR, odds ratio; OS, overall survival; SD, standard deviation; SEER, Surveillance, Epidemiology, and End Results; SVI, Social Vulnerability Index; TO, textbook outcome; USDA, US Department of Agriculture.

## Data Availability

No new data were created or analyzed in this study. Data sharing is not applicable to this article.
